# The Role of Cardiovascular Magnetic Resonance Imaging in Patients with Cardiac Arrhythmias

**DOI:** 10.31083/j.rcm2409252

**Published:** 2023-09-15

**Authors:** Chrysovalantou Nikolaidou, Julian O.M. Ormerod, Antonios Ziakas, Stefan Neubauer, Theodoros D. Karamitsos

**Affiliations:** ^1^Oxford Centre for Clinical Magnetic Resonance Research, University of Oxford, John Radcliffe Hospital, Headington, OX3 9DU Oxford, UK; ^2^Division of Cardiovascular Medicine, Radcliffe Department of Medicine, University of Oxford, John Radcliffe Hospital, Headington, OX3 9DU Oxford, UK; ^3^First Department of Cardiology, AHEPA Hospital, School of Medicine, Faculty of Health Sciences, Aristotle University of Thessaloniki, 54636 Thessaloniki, Greece

**Keywords:** Cardiovascular magnetic resonance, cardiac arrhythmias, high-quality CMR imaging, cardiomyopathy

## Abstract

Cardiac arrhythmias are associated with significant morbidity, mortality and poor quality of life. Cardiovascular magnetic resonance (CMR) imaging, with its unsurpassed capability of non-invasive tissue characterisation, high accuracy, and reproducibility of measurements, plays an integral role in determining the underlying aetiology of cardiac arrhytmias. CMR can reliably diagnose previous myocardial infarction, non-ischemic cardiomyopathy, characterise congenital heart disease and valvular pathologies, and also detect the underlying substrate concealed on conventional investigations in a significant proportion of patients with arrhythmias. Determining the underlying substrate of arrhythmia is of paramount importance for treatment planning and prognosis. However, CMR imaging in patients with irregular heart rates can be problematic. Understanding the different ways to overcome the limitations of CMR in arrhythmia is essential for providing high-quality imaging, comprehensive information, and definitive answers in this diverse group of patients.

## 1. Introduction

Cardiac arrhythmias are associated with significant morbidity, mortality and poor health-related quality of life. Atrial fibrillation (AF) is the most common cardiac arrhythmia and is expected to affect 12.1 million people in the United States in 2030 and 17.9 million people in the European Union by 2060 [1]. Ventricular arrhythmias (VAs) encompass a wide spectrum of abnormal cardiac rhythms, ranging from asymptomatic premature ventricular contractions (PVCs) to sustained ventricular tachycardia (VT), ventricular fibrillation and sudden cardiac death (SCD) [2]. VAs are thought to be responsible for 75% to 80% of cases of SCD [3]. PVCs are the most common VA in the general population and can be found in 40% to 75% of individuals on 24- to 48-hour Holter monitoring [4]. While they often exhibit a benign behaviour, frequent PVCs can be associated with ventricular dysfunction or may indicate underlying structural heart disease, which may be concealed on conventional diagnostic investigations in a substantial proportion of patients [5].

Cardiovascular imaging plays an integral role in determining the underlying etiology of arrhythmias, detecting their adverse effects on cardiac structure and function, and guiding treatment [6]. Specifically, cardiac magnetic resonance (CMR) provides accurate and reproducible assessment of cardiac morphology and function, enabling detailed myocardial tissue characterisation with a high degree of precision. However, CMR imaging in patients with irregular heart rates can be problematic. This review will focus on the role of CMR in the diagnosis, prognostication, and treatment planning of patients with cardiac arrhythmias. Additionally, ways to ensure high quality imaging despite the technical challenges posed by arrhythmia will be discussed.

## 2. Determining the Underlying Etiology of Arrhythmia

Determining the underlying substrate of arrhythmia is paramount for treatment planning and prognosis. A significant number of patients may exhibit elements of underlying heart disease on CMR even when results from other imaging tests, including echocardiography, are normal [7, 8]. The presence of regional or diffuse myocardial fibrosis can be readily detected on late gadolinium enhancement (LGE) and parametric mapping, respectively, enabling a better determination of the underlying etiology of arrhythmia. Conversely, a normal CMR scan provides relative reassurance for the majority of patients, allowing the focus to shift to arrhythmia causes unrelated to myocardial disease.

### 2.1 Ischaemic Heart Disease

**Fig. 1.**
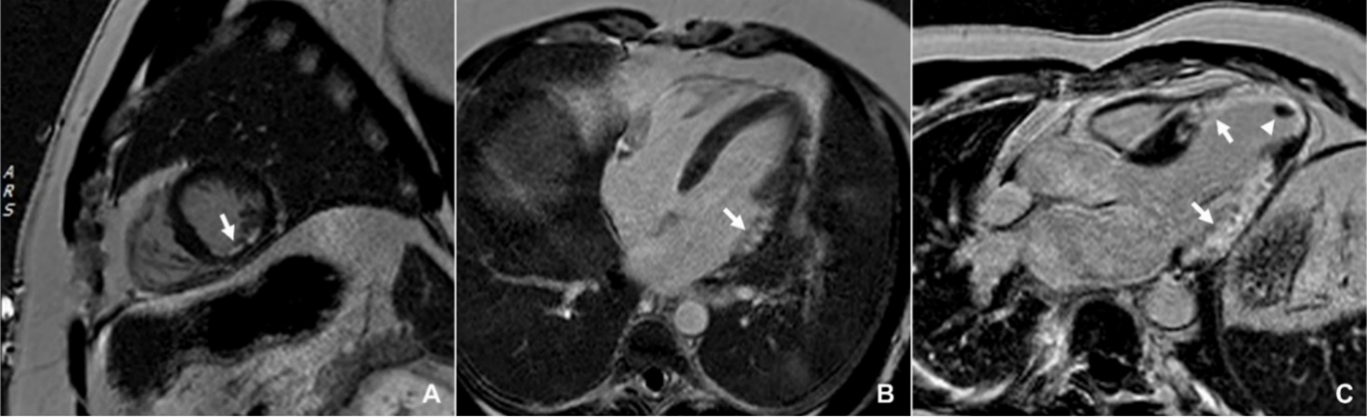
**Late gadolinium enhancement pattern in myocardial infarction**. Different extent of myocardial infarction on phase-sensitive inversion recovery (PSIR) late gadolinium enhancement images. (A) mid-ventricular short axis slice showing a subendocardial infarction, ∼50% wall thickness in the mid-inferior wall (arrow); (B) horizontal long axis view showing a subendocardial infarction, ∼75% wall thickness in the basal anterolateral wall (arrow); (C) left ventricular outflow tract view showing two large transmural myocardial infarctions, one involving the anteroseptum and apex and one affecting the inferolateral wall (arrows), with an apical thrombus (arrowhead).

Previous myocardial infarction (MI) is the most common substrate for cardiac arrhythmias, especially VAs. CMR can provide an accurate and reproducible assessment of cardiac volumes and left ventricular ejection fraction (LVEF) and reliably demonstrate the extent of MI on LGE, with higher sensitivity compared to other imaging techniques (Fig. 1). LVEF is considered the most reliable predictor of future cardiac events and cardiac arrhythmias in patients with ischaemic heart disease [9]. However, the LVEF showed only moderate performance in predicting SCD in patients with previous MI in the phase one analysis of the multicentre PROFID (Prevention Of Sudden Cardiac Death After Myocardial Infarction By Defibrillator Implantation) project [10, 11].

The presence and spatial extent of MI on LGE have been shown to predict mortality and major adverse cardiac events (MACE) in patients with coronary artery disease, beyond and independently of LVEF and left ventricular (LV) volumes [12]. As a result, they may serve as more reliable predictors of VA inducibility than LVEF [13] and can be used, together with electrocardiographic criteria and programmed ventricular stimulation, to guide treatment with an implantable cardioverter-defibrillator (ICD) in patients with previous MI and preserved LVEF [14]. The infarct size on CMR is associated with all-cause mortality, but the data regarding the role of MI extent or MI border zone in predicting arrhythmic outcomes and SCD are discordant and inconclusive [10, 15, 16].

### 2.2 Dilated Cardiomyopathy

**Fig. 2.**
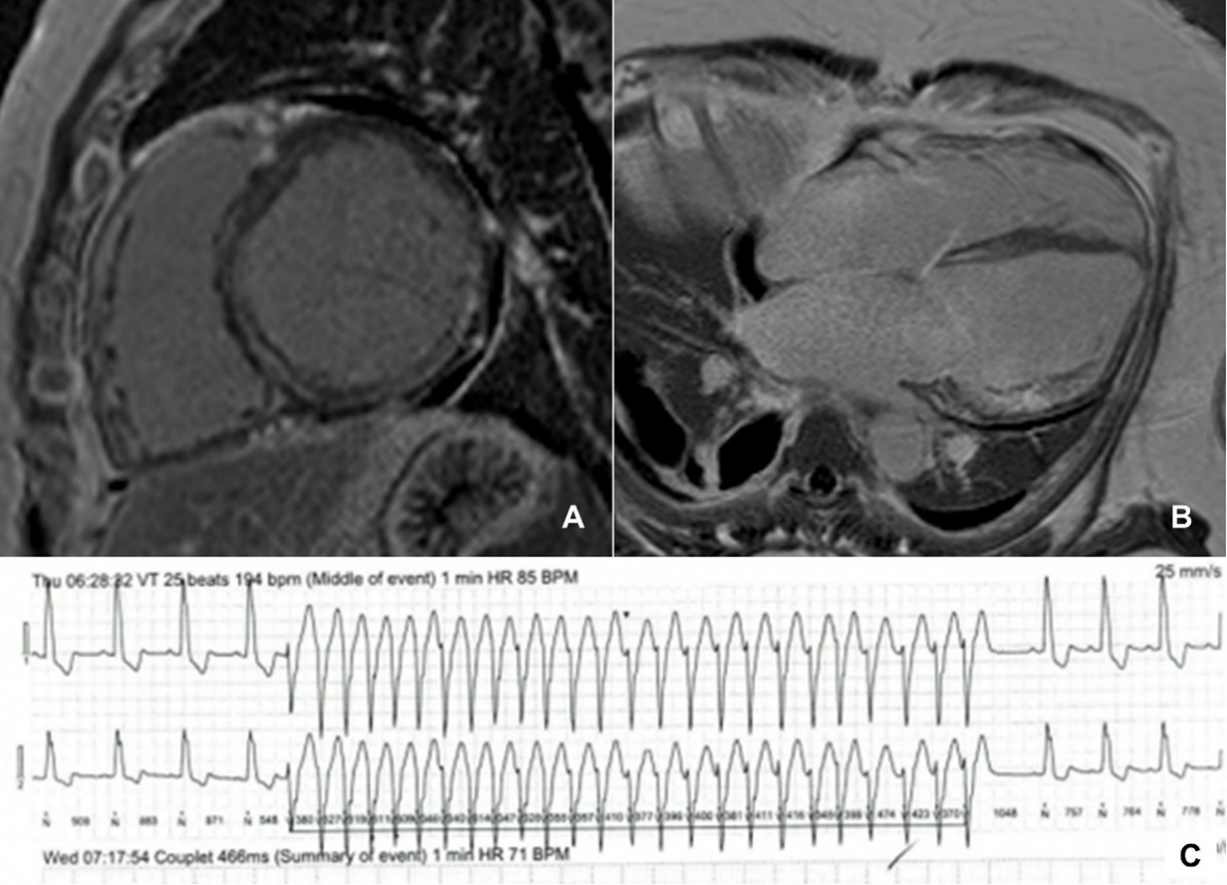
**Late gadolinium enhancement pattern in dilated cardiomyopathy**. Mid-ventricular short-axis (A) and horizontal long axis (B) late gadolinium enhancement images in a patient with dilated cardiomyopathy, severely dilated left ventricle and extensive mid-wall fibrosis. (C) monomorphic non-sustained ventricular tachycardia on an electrocardiogram rhythm strip from the same patient. BPM, beats per minute; HR, heart rate; VT, ventricular tachycardia.

Dilated Cardiomyopathy (DCM) patients suffer from a significant burden of atrial and ventricular arrhythmias, which are often poorly tolerated due to LV systolic dysfunction [17]. The presence of fibrosis on LGE, detected in approximately one-third of patients with DCM (Fig. 2), strongly predicts adverse cardiac events, all-cause mortality, heart failure hospitalisation, heart transplantation, as well as arrhythmic events, ICD implantation, and SCD, beyond LVEF [18, 19]. A subgroup analysis of the DANISH (Defibrillator Implantation in Patients with Nonischemic Systolic Heart Failure) trial in a small number of DCM patients showed that LGE predicts all-cause mortality but did not show improved survival with ICD treatment [20]. However, this analysis was not powered to show a difference in subgroups. Importantly, LGE can predict sustained VAs, appropriate ICD therapy, SCD, and aborted SCD across a wide spectrum of DCM patients, even in those with moderate or mild LV systolic dysfunction, for whom the risk of SCD is unclear [21, 22]. The ongoing ReCONSIDER (Arrhythmic Risk Stratification in Nonischemic Dilated Cardiomyopathy) study aims to evulate a two-step multifactorial approach, including CMR and programmed electrical stimulation, in the risk stratification of DCM patients with either relatively preserved or reduced systolic function [23]. Finally, prolonged native myocardial T1 values and elevated extracellular volume (ECV) in DCM patients in the context of diffuse fibrosis, not detectable on LGE, have also been associated with an increased risk of VA and MACE [24, 25] and can potentially improve risk stratification [26].

The term left ventricular non-compaction (LVNC) has been used in cases of LV cardiomyopathy with pronounced trabeculation. Current evidence suggests that the term is inaccurate, and ‘excessive trabeculation’ should be used instead. It can be associated with underlying cardiomyopathy or represent a normal variant, such as in athletes and pregnant women. The risk of arrhythmia, SCD, or other adverse cardiac events is determined by the underlying cardiomyopathy, increased age, increased LV size, LV dysfunction, and symptomatic heart failure [27]. The presence of hypertrabeculation does not alter the prognosis, so the need for a primary prevention ICD is in general guided by the severity of LV systolic dysfunction [28, 29]. However, LGE, which is detected only in a small proportion of patients seems to be associated with MACE and a worse prognosis [30].

### 2.3 Hypertrophic Cardiomyopathy

Hypertrophic Cardiomyopathy (HCM) is the most common hereditary cardiomyopathy [31]. VAs, particularly non-sustained ventricular tachycardia (NSVT), are a common finding in patients with HCM [32]. SCD is the most devastating complication, often affecting young and frequently asymptomatic patients. NSVT is more frequently recorded on Holter monitoring with increasing hypertrophy and has been shown to be associated with SCD.

**Fig. 3.**
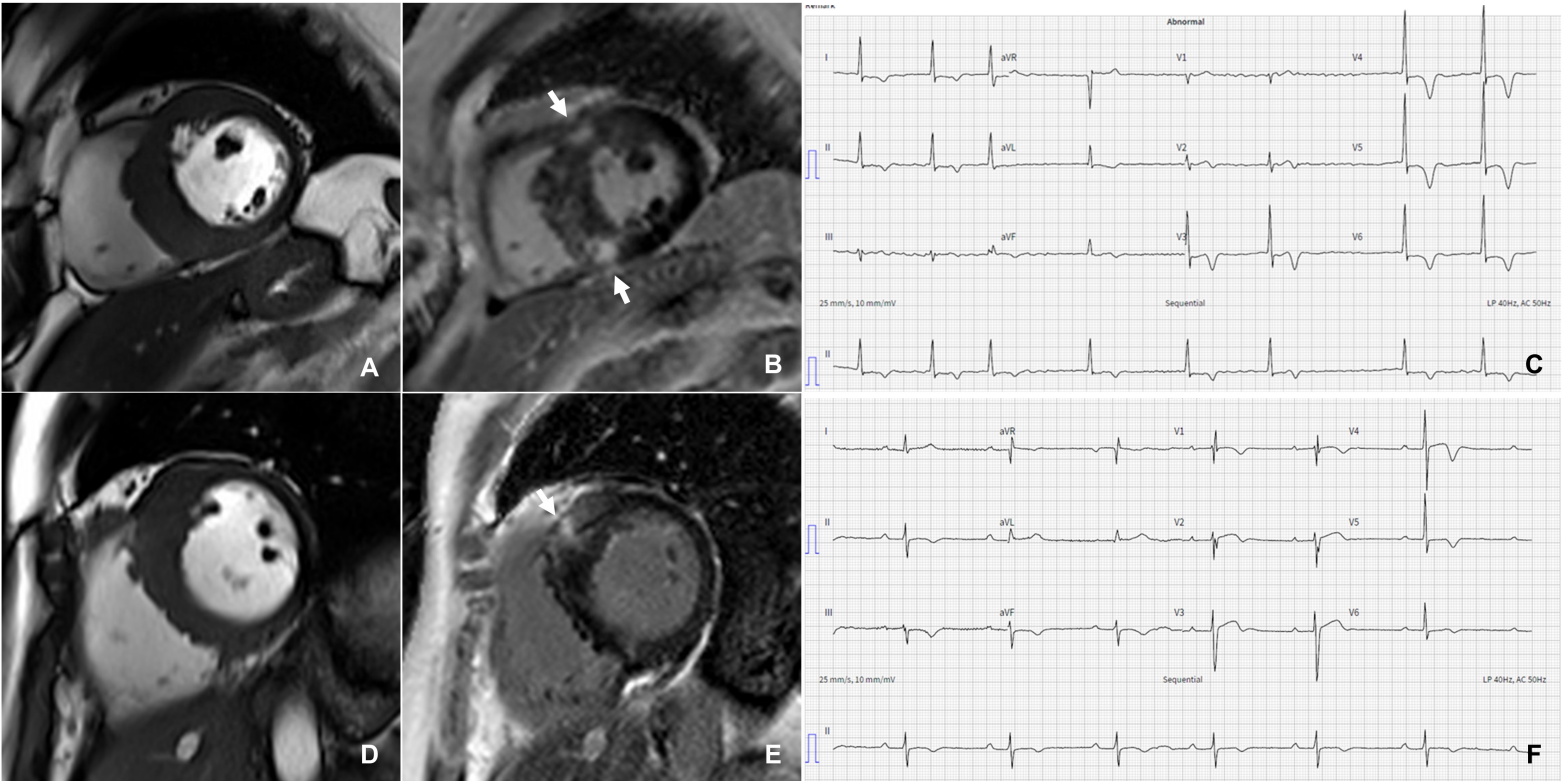
** Types of hypertrophy and late gadolinium enhancement patterns in hypertrophic cardiomyopathy**. Severe asymmetrical septal hypertrophy (A), with extensive patchy and hazy LGE in the septum and both right ventricular insertion points (arrows) (B) in a patient with hypertrophic cardiomyopathy and atrial fibrillation (C). Severe anteroseptal hypertrophy (D) with patchy and hazy LGE around the anterior right ventricular insertion point (arrow) (E) in a patient with hypertrophic cardiomyopathy and first-degree atrioventricular block (F). LGE, late gadolinium enhancement.

Patients with HCM demonstrate LGE in 50–80% of cases, depending on the selection criteria [33]. The pattern of LGE is diverse but usually involves patchy and/or hazy mid-wall enhancement and enhancement in the right ventricular septal insertion points (Fig. 3). LGE is considered a marker of replacement fibrosis, which may represent the substrate for arrhythmia, and has been associated with increased all-cause and cardiac mortality, progression to end-stage HCM, heart failure admissions, sustained VT or ventricular fibrillation, or appropriate ICD discharge [34]. Extensive LGE (≥15% of myocardial mass) can identify patients at increased risk of SCD and can increase the discriminative power of risk prediction models [35]. The current HCM SCD risk prediction model of the European Society of Cardiology (ESC) uses seven clinical parameters to estimate the 5-year risk of SCD, including NSVT and maximal LV wall thickness [36], which can be under- or overestimated with echocardiography but can be precisely and reliably measured on CMR. Although the ESC HCM risk prediction tool has been validated in a diverse cohort of patients with HCM [37], there is still room for improvement, especially in intermediate risk patients and patients without the conventional high-risk features. The US guidelines recommend consideration of several factors that are absent in the ESC risk calculator, including the presence of LV dysfunction, LV apical aneurysm, or widespread LGE in the absence of other risk factors [38]. Real world data indicate that the American College of Cardiology/American Heart Association 2020 guidelines had high sensitivity and negative predictive value but showed modest specificity for SCD, in comparison to the ESC HCM risk score, which showed higher specificity but lower sensitivity [39].

Fractional anisotropy is a novel *in vivo* marker of myocardial disarray in patients with HCM, measured using diffusion tensor CMR. A recent study showed that low fractional anisotropy is associated with increased risk of VAs and can be potentially used as an additional risk factor for VAs in patients with HCM [40]. The association of T1-mapping and ECV with adverse outcomes in patients with HCM is under investigation [41], with a recent study showing positive correlation of global native T1 mapping with MACE [42]. The current National Heart, Lung, and Blood Institute (NHLBI)-Hypertrophic Cardiomyopathy Registry study (HCMR) is evaluating the role of LGE, T1 mapping, ECV, and other CMR parameters together with genetics and serum biomarkers in predicting clinical events in 2755 HCM patients [43], and outcome results are expected in 1–2 years.

AF is the most common arrhythmia and a major risk factor for stroke in HCM patients, with a reported prevalence of ~20–30% and an incidence of de-novo AF of approximately 2% per year [44]. Increased left atrial (LA) volumes and low LA ejection fraction measured on CMR have been proven as strong determinants of AF in patients with HCM [45]. More specifically, in the HCMR trial, increased LA volume and reduced LA contractile percent were among the major predictors of AF endpoints, such as electrical cardioversion, catheter ablation, hospitalization, or decision to accept permanent AF [46]. Furthermore, LA LGE is more common in HCM patients with AF [47] and is associated with an increased rate of new-onset atrial arrhythmia [48]. The extent of LGE of the myocardium also significantly correlates with AF but is inferior to the LA size [45]. A recent study showed that LA strain components measured by CMR are impaired in HCM patients and can independently predict the risk of new-onset AF [49].

### 2.4 Arrhythmogenic Cardiomyopathy

**Fig. 4.**
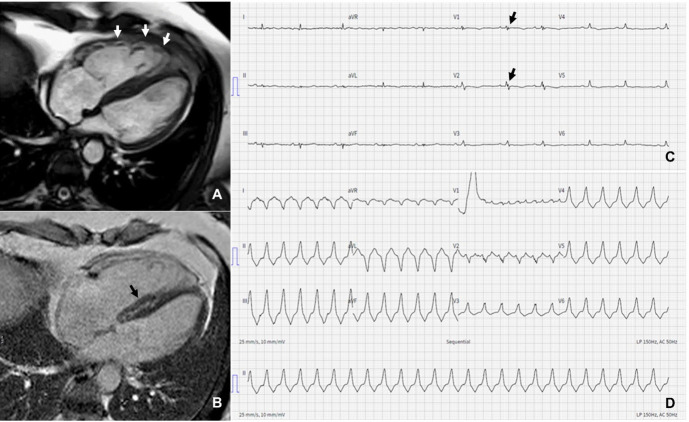
**CMR in a patient with arrhythmogenic cardiomyopathy**. (A) The right ventricle is dilated with dyskinetic areas in systole (white arrows) on the horizontal long axis view. (B) Late gadolinium enhancement in both the right ventricular free wall and ventricular septum (black arrow). (C) T-wave inversion in leads V1–V4 and epsilon waves (black arrows) on baseline electrocardiogram. (D) Three months after the CMR scan the patient presented with sustained ventricular tachycardia. CMR, cardiac magnetic resonance.

Arrhythmogenic Cardiomyopathy (ACM), formerly known as arrhythmogenic right ventricular cardiomyopathy (ARVC), causes regional and/or global ventricular dysfunction and predisposes to life-threatening VAs, such as monomorphic or polymorphic VT or ventricular fibrillation, which can lead to SCD, even in young and apparently healthy individuals. Risk stratification in ACM is challenging. Major risk factors for sudden death include sustained VA (especially if poorly tolerated), significant dysfunction of one or both ventricles (left or right ventricular ejection fraction <35–40%) and presumed arrhythmic syncope [50]. CMR can play an important role in the prognostic stratification of patients with ACM by identifying ventricular systolic dysfunction, the presence of aneuryms, an increased amount of fibro-fatty infiltration and LGE, and a higher fat-to-LGE ratio, which have been shown to correlate with worse prognosis [51, 52]. LGE is usually transmural in the thin right ventricular (RV) walls, while in patients with LV involvement there is subepicardial to mid-wall enhancement, most commonly seen in the lateral LV wall and/or septum [52, 53] (Fig. 4). Furthermore, myocardial T1-mapping can detect diffuse fibrosis even in patients with no LGE and offers the potential for early detection of LV involvement in patients with ACM or first-degree relatives at risk [54]. Moreover, impaired myocardial strain assessed with CMR can potentially aid in the early diagnosis of ACM [55] and improve the detection of arrhythmogenic VT substrate [56].

### 2.5 Cardiac Amyloidosis

**Fig. 5.**
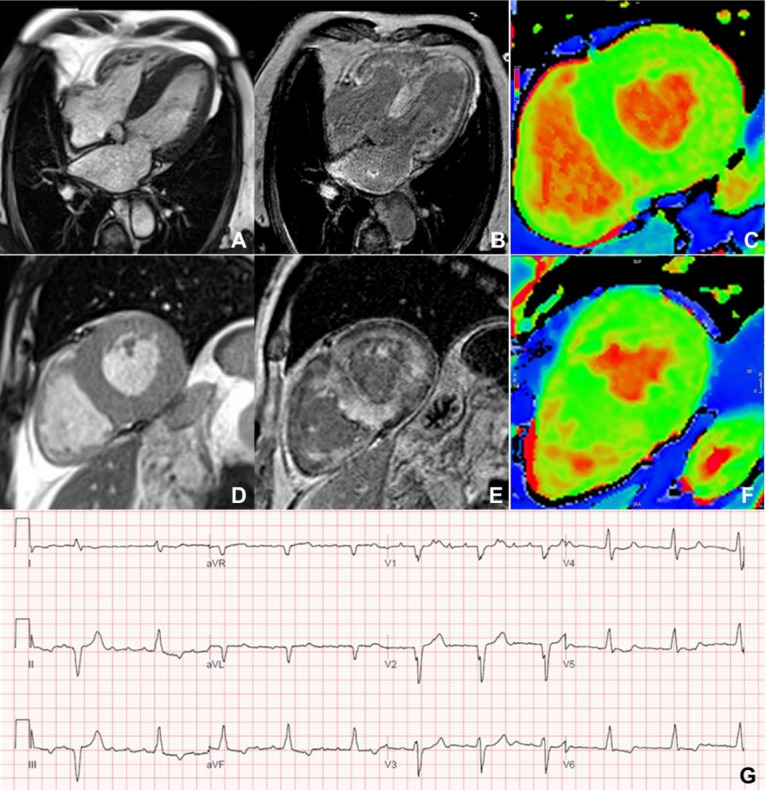
**CMR in a patient with cardiac amyloidosis**. (A,D) Horizontal long-axis and mid-ventricular short-axis SSFP images showing concentric left ventricular hypertrophy. (B,E) Late gadolinium enhancement images showing extensive subendocardial enhancement, enhancement of right ventricular and atrial walls, and the characteristic appearance of a dark blood pool. (C,F) mid-ventricular short-axis images on ShMOLLI T1-mapping, demonstrating multiple areas with increased T1 values (yellow/red areas within the myocardium) due to the deposition of amyloid fibrils. (G) The patient presented with atrial fibrillation and complete heart block. CMR, cardiac magnetic resonance; SSFP, steady-state free precession.

The most common arrhythmia in patients with cardiac amyloidosis is AF [57], but bradycardia or heart block are also commonly seen. VAs and SCD are frequent modes of death, and anecdotally, VAs are particularly common upon induction of chemotherapy treatment for amyloid light-chain (AL) amyloidosis [58]. CMR has emerged as a valuable tool in the diagnosis of cardiac amyloidosis by providing non-invasive tissue characterisation. The characteristic pattern on LGE, typically (though not always) due to substantial interstitial expansion with amyloid, demonstrates global subendocardial enhancement, which, in cases of involvement of the RV endocardium, gives rise to the so-called ‘Zebra sign’ [59]. There may also be areas of mid-wall/transmural enhancement. Another characteristic on CMR is the abnormal myocardial and blood-pool gadolinium kinetics, resulting in difficulty nulling the myocardium together with a dark blood pool [60] (Fig. 5). Native myocardial T1 values on T1-mapping and ECV are significantly elevated in patients with cardiac amyloidosis and are useful not only in the diagnosis of the disease, especially in the early stages of cardiac involvement, but also in predicting adverse cardiac events and survival [61, 62]. The presence of myocardial oedema in untreated patients with cardiac amyloidosis, as demonstrated by elevated T2 values on T2-mapping, is associated with worse survival [63]; the correlation with arrhythmic events needs further investigation.

### 2.6 Inflammatory Cardiomyopathies

**Fig. 6.**
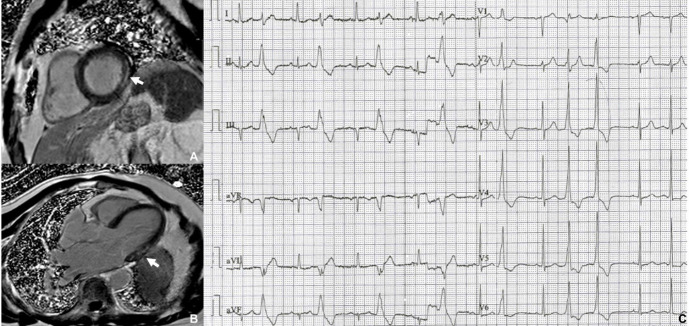
**Late gadolinium enhancement pattern in myocarditis**. Subepicardial/mid-wall enhancement in the inferolateral left ventricular wall (arrows) on short-axis and 3-chamber late gadolinium enhancement images (A, B) in a patient with previous myocarditis and frequent ventricular ectopics (C).

Myocarditis may be associated with cardiac arrhythmia either during the acute (“hot”) inflammatory phase in approximately 25% of patients, or during the chronic post-inflammatory stage. Cardiac arrhythmias can vary, from supraventricular arrhythmias in patients with minimal myocardial inflammation and AF commonly in patients with isolated atrial giant cell myocarditis, to advanced atrioventricular block, VAs and SCD [64]. Different autopsy series report highly variable prevalence of myocarditis, ranging from 2% to 42% in young people with SCD [65]. Importantly, undiagnosed previous or chronic myocarditis can present as unexplained PVCs in otherwise healthy individuals [66]. CMR can identify active or previous myocarditis in patients with unexplained VAs by demonstrating acute myocardial eodema on T2-weighted imaging, hyperaemia on early gadolinium imaging, and/or scar on LGE, as described in the Lake Louise criteria for the non-invasive diagnosis of myocarditis [67]. CMR is indicated in patients with new-onset or persisting symptoms, evidence of significant myocardial injury, and suspected viral aetiology [68]. The enhancement pattern on LGE is usually subepicardial to mid-wall, often in a patchy distribution, commonly localized in the inferolateral and, less frequently, in the anteroseptal segments of the LV, and can extend to a variable degree throughout the ventricular wall. However, the subendocardium is characteristically spared [67] (Fig. 6). In 2018, the Lake Louise diagnostic criteria were updated to include T2 and T1 mapping for the identification of myocardial inflammation [69]. The presence of LGE on CMR may be helpful in the risk prognostication of patients with myocarditis. Septal location and mid-wall or patchy LGE have demonstrated the strongest associations with MACE [68, 70]. Conversely, patients with myocarditis and a normal CMR scan have a good prognosis independent of their clinical symptoms [71].

**Fig. 7.**
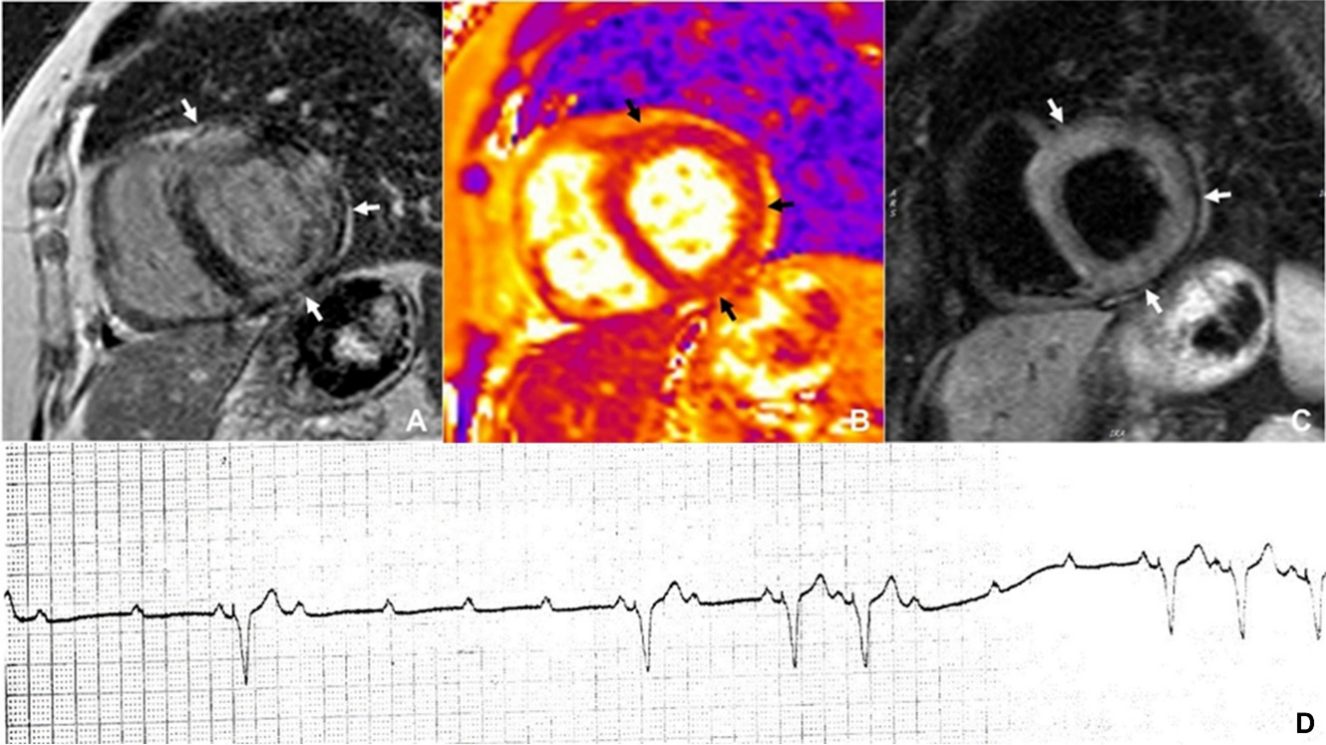
**CMR imaging in a patient with cardiac sarcoidosis**. Large patches of mid-wall late gadolinium enhancement in the anterior, inferolateral and inferior left ventricular walls (white arrows) (A) in a patient with cardiac sarcoidosis and episodes of complete heart block (D). Co-localized myocardial edema seen as brighter areas on T2-mapping (B; black arrows) and T2-weighted images (C; white arrows). CMR, cardiac magnetic resonance.

Patients with cardiac sarcoidosis are at increased risk of VAs, conduction abnormalities, and SCD, which sometimes require implantation of a pacemaker or an ICD. VAs are a significant predictor of mortality [72], with areas of scar/fibrosis and possibly myocardial inflammation within the left or right ventricle considered as the dominant substrates [73]. Atrial arrhythmias can occur in up to one quarter of patients with cardiac sarcoidosis, with granulomatous involvement of the atria and/or LA dilatation and elevated atrial pressures as the potential aetiologies [74]. CMR in patients with cardiac sarcoidosis can detect wall motion abnormalities and areas of thinning, ventricular dysfunction, as well as the presence of myocardial oedema or scarring. LGE is usually patchy, subepicardial and/or mid-wall, but can also be transmural, along the basal septum and/or inferolateral LV wall [75] (Fig. 7). The presence of LGE is associated with a significantly higher risk of future adverse cardiac events, such as VAs, appropriate ICD discharge, pacemaker implantation and cardiac death, compared to patients without LGE, independent of LVEF and LV end-diastolic volume [75, 76, 77].

### 2.7 Valvular Heart Disease

Mitral valve prolapse (MVP) is a common structural abnormality of the mitral valve, affecting 2% to 3% of the general population [78]. A proposed separate phenotype of MVP, causing VA and SCD in the absence of valve failure - so called “arrhythmic MVP” - has been advocated since before the advent of two-dimensional (2D) echocardiography [79], but remains controversial. Fibrosis in the papillary muscles and inferobasal wall, mitral annulus disjunction, and systolic curling of the posterior leaflet, as described by pathological and CMR studies, have been linked to more frequent cardiac arrhythmias and increased arrhythmic risk in patients with MVP, even in the absence of severe mitral regurgitation [80, 81]. Furthermore, increased ECV of the basal LV segments may be an additional marker of increased arrhythmic risk in patients with MVP, even in patients with no LGE [82].

Aortic valve stenosis is the most common primary valve lesion requiring intervention in the Western world. During the course of the disease, there is progression from reactive diffuse interstitial fibrosis, which can be detected on T1-mapping and ECV quantification, to replacement fibrosis detected on LGE [83]. Replacement fibrosis is predictive of all-cause and cardiac mortality [84] and can potentially improve risk stratification and patient selection for early intervention in severe aortic stenosis.

### 2.8 Congenital Heart Disease

**Fig. 8.**
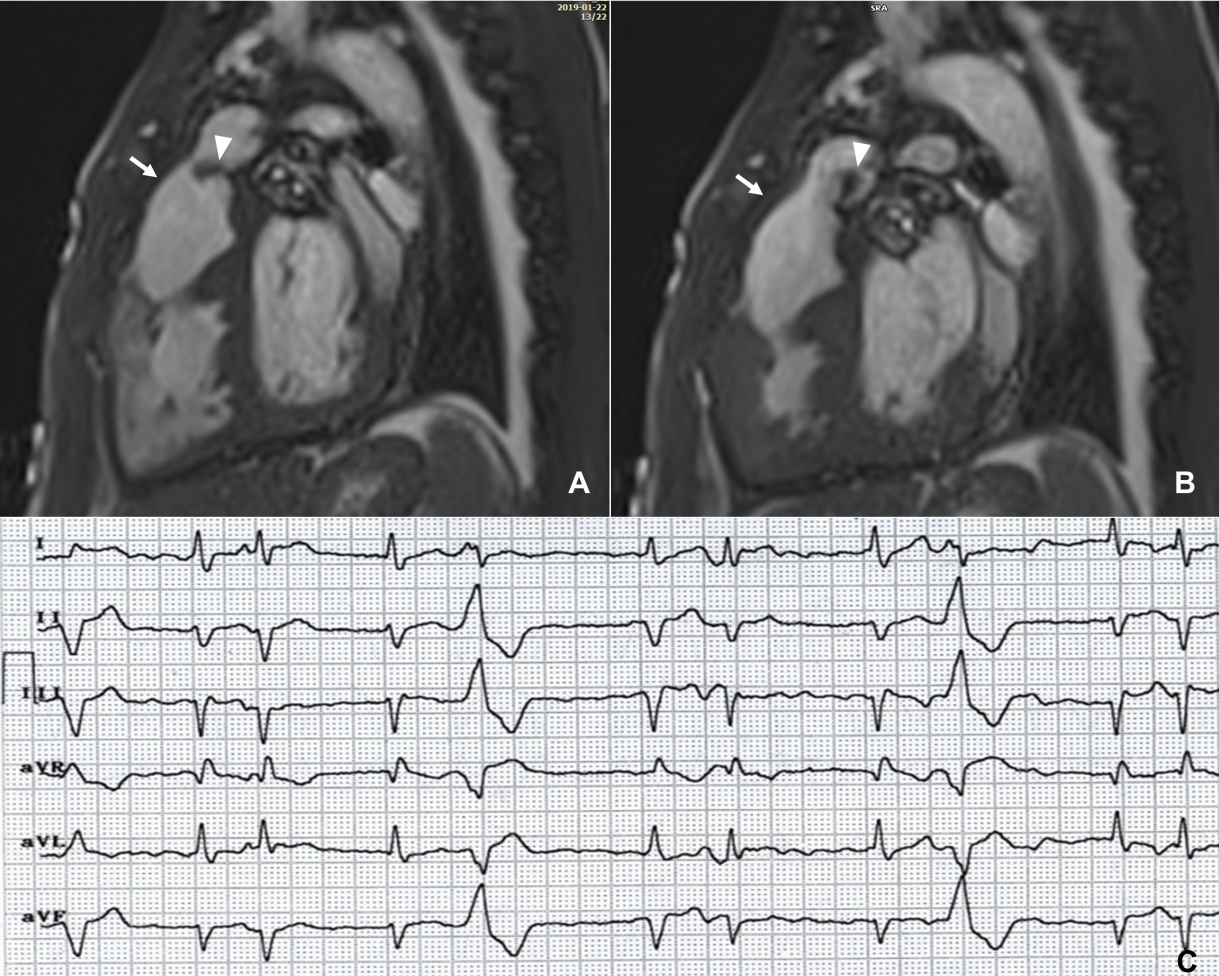
**CMR imaging in a patient with repaired tetralogy of Fallot**. Vegetation on the pulmonary valve homograft (arrowhead) seen in diastole (A) and in systole (B). The white arrow shows the RVOT patch repair. The patient presented with frequent premature supraventrilucar and ventricular contractions (C). RVOT, right ventricular outflow tract. CMR, cardiac magnetic resonance.

Patients with congenital heart disease suffer from arrhythmias due to the underlying heart defect, genetic influences, or as a result of surgical or interventional treatment [85]. Risk stratification for VAs and SCD in the growing population of patients with adult congenital heart disease remains challenging. Patients with tetralogy of Fallot, transposition of the great arteries after atrial switch surgeries, cyanotic heart disease, Ebstein anomaly, and Fontan circulation have higher risk substrates for SCD [86]. A detailed description of the role of CMR in congenital heart disease is beyond the scope of this review. Briefly, CMR can demonstrate the complex cardiac anatomy, improve arrhythmic risk stratification, and helps guide electrophysiology procedures. In patients with repaired tetralogy of Fallot in particular (Fig. 8), the RV and LV ejection fraction measured on CMR and the extent of LGE of either or both ventricles have been incorporated into a risk score. Together with clinical parameters and brain natriuretic peptide (BNP) levels, this score can identify the subgroup of patients with high annual mortality risk [87].

### 2.9 Structurally Normal Hearts

Excluding underlying heart disease with the gold-standard test of CMR can provide very important diagnostic and prognostic information in patients with arrhythmias. For example, a normal CMR in patients presenting with aborted cardiac arrest allows the focus to shift to identifying channelopathies, which have specific treatments and are commonly heritable. Furthermore, in patients presenting with haemodynamically tolerated sustained monomorphic VT (so called “normal heart VT”), a normal CMR puts them into a dramatically better prognostic group, as SCD in this context is rare [88]. Conversely, CMR with tissue characterisation can detect underlying substrate concealed on conventional investigations in a significant proportion of patients with idiopathic VAs, such as PVCs, which varies from 13.7% to 78.8% according to the study population. Additionally, myocardial strain evaluated with CMR is more sensitive than LVEF in detecting incipient contractile dysfunction in patients with frequent idiopathic VAs and PVC-related or other types of underlying cardiomyopathy, and can thus guide early treatment [66]. Importantly, myocardial structural abnormalities detected on CMR can redefine patient prognosis and improve risk stratification, as they are associated with worse clinical outcomes, such as nonfatal episodes of ventricular fibrillation or sustained VT, resuscitated cardiac arrest, or SCD [89, 90].

## 3. Acquiring Good Quality CMR Images in Patients with Arrhythmia

Cardiac motion during CMR scanning constitutes a major source of image degradation and artefacts. This is accounted for by electrocardiogram (ECG)-gated image acquisition, synchronized to the patient’s heart rate throughout the cardiac cycle, and then image reconstruction from the information acquired over several heart beats. For cine imaging, cardiac synchronization is performed either with retrospective ECG gating or with prospective ECG triggering until nearly at the end of the cardiac cycle, leaving a small “blind spot” at end-diastole to facilitate the capture of signal starting from the next R wave. While retrospective gating works well for small variations in the RR interval, when the heart rate is irregular, beat-to-beat variations in the cardiac cycle length cause artefacts during image reconstruction [91] (**Supplementary Video 1**).

**Fig. 9.**
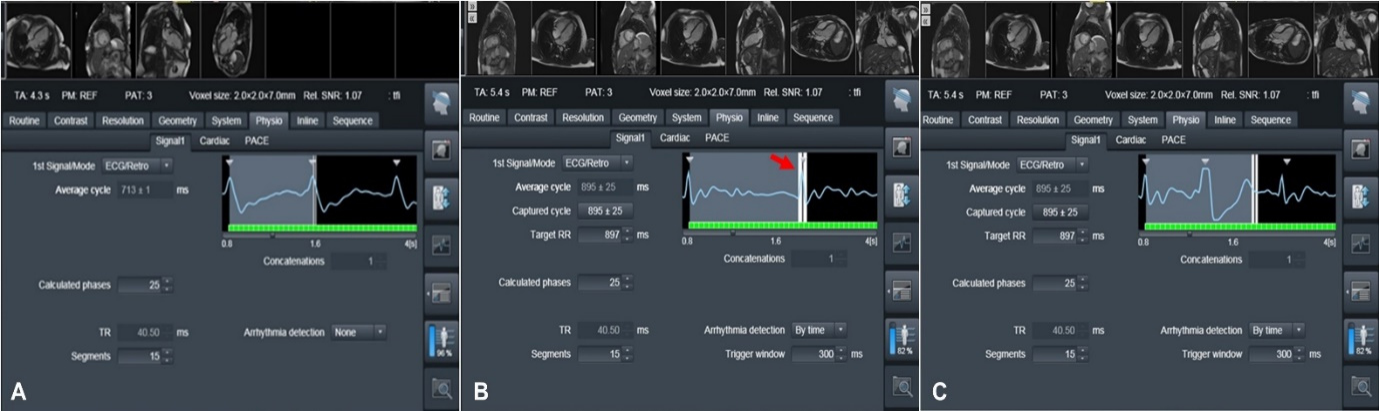
**ECG gating with and without arrhythmia detection**. (A) retrospective ECG gating in a patient with sinus rhythm. (B,C) With arrhythmia detection, data are acquired from cycles where the R wave falls during the RR interval and trigger window set by the operator (red arrow), while data during any other interval are ignored (C). ECG, electrocardiogram.

In patients with occasional ectopic beats, arrhythmia rejection algorithms can be used to acquire good quality cine images. The technique involves retrospectively gating the whole cardiac cycle while setting an optimum RR interval that is designed to ignore the data acquired during any other interval that falls outside this set one (Fig. 9). However, in patients with frequent arrhythmias and grossly irregular rhythm, it is impossible to predict an optimal trigger window, and therefore the rejection of a large amount of data leads to significantly longer breath-hold times [91]. In this setting, a safe and effective alternative approach to suppress the PVCs and acquire high-quality images is the bolus intravenous administration of an anti-arrhythmic medication, such as procainamide [92]. In cases with frequent PVCs or AF, prospective triggering may provide diagnostic image quality (**Supplementary Video 2**). The acquisition window must be set short enough to exclude the ectopic beat or slightly shorter than the shortest RR interval in AF. The main disadvantage of the technique is the acquisition of an incomplete cardiac cycle missing out the end-diastole in longer RR intervals, resulting in low temporal resolution [93].

**Fig. 10.**
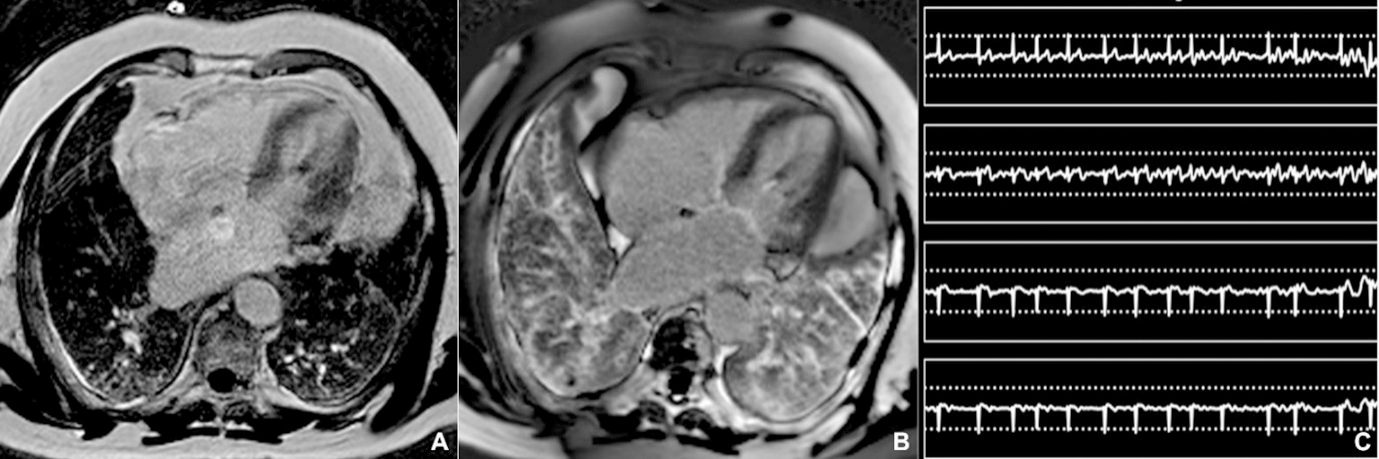
**Motion-corrected LGE imaging**. (A) Impaired quality on horizontal long axis breath-hold LGE image, which is significantly improved using a motion-correction free-breathing LGE sequence (B) in a patient with atrial fibrillation (C). LGE: late gadolinium imaging.

Retrospectively gated radial acquisitions can potentially improve image quality, as the noise produced by an ectopic beat is radiated across the entire image. However, technical characteristics of the sequence and other forms of imperfections can cause significant artefacts in the final image, especially in patients with a high burden of arrhythmia [94]. Evolving techniques such as the Extra-Dimensional Golden-angle Radial Sparse Parallel Imaging (XD-GRASP) and the two-step 2D filtered back-projection 3D radial data reconstruction can potentially improve the reconstruction speed and reduce motion artefacts. Real-time cine imaging enables fast data collection without the need for breath-holding; however, at the expense of temporal resolution. View sharing is another commonly used technique that accelerates image acquisitions and can provide diagnostic image quality (**Supplementary Video 3**). Another technique offering the potential to image arrhythmic patients is compressed sensing, which provides a full short-axis stack in a single breath-hold with fewer heartbeats than any conventional cine imaging [95]. The resulting image quality with real-time imaging and the accelerated image acquisition techniques is diagnostic but slightly lower than with standard retrospective ECG-gated breath-hold cine imaging (**Supplementary Videos 4,5**).

Finally, motion-corrected free-breathing LGE sequences can provide high quality CMR images, easier to interpret, with potentially shorter acquisition times than with conventional breath-held acquisition [96]. This can be advantageous in the setting of cardiac arrhythmias, such as AF and frequent premature supraventricular or ventricular contactions (Fig. 10). The different techniques used to improve image quality in arrhythmia patients are presented in Table 1.

**Table 1.** **Strategies to to improve image quality in patients with different types of arrhythmia**.

Techniques used for improving CMR image quality	Type of arrhythmia
Prospective ECG gating	∙ Atrial fibrillation
	∙ Premature contractions
	∙ Respiratory sinus arrhythmia
Arrhythmia rejection algorithms	Infrequent premature supraventricular or ventricular contractions
View sharing	
Real-time cine imaging	∙ Atrial fibrillation
Compressed sensing	∙ Premature contractions
Retrospectively gated radial acquisition	Infrequent premature ventricular contractions
Pharmacological suppression	Frequent premature ventricular contractions
Motion-corrected LGE imaging	All types of supraventricular or ventricular arrhythmia

CMR, cardiac magnetic resonance; ECG, electrocardiogram; LGE, late gadolinium enhancement.

## 4. Novel Techniques and Future Perspectives

As already described, parametric mapping and ECV can detect myocardial scarring or fibrosis, especially in cases with diffuse fibrosis not detectable on LGE. They can help identify potential substrates for VAs, improve risk stratification and guide treatment (17, 37). Importantly, emerging technologies such as virtual LGE can transform native T1 maps into images resembling conventional LGE images, allowing faster scanning without the need for contrast administration [97]. Diffusion tensor cardiac magnetic resonance is a novel imaging technique that can show myocardial microstructure by mapping the diffusion of water molecules. Findings on diffusion sensor CMR have been shown to be predictive of the risk of VA in patients with HCM [40] and can potentially differentiate between different cardiomyopathy phenotypes [98]. In terms of image quality, artificial intelligence and deep learning show promise in motion detection and modeling, as well as faster image reconstruction, which can significantly enhance image quality even in patients with arrhythmia [99, 100].

## 5. Conclusions

CMR has emerged as a powerful non-invasive imaging tool that can determine the underlying aetiology and guide treatment in patients with cardiac arrhythmias. Importantly, CMR can help in the risk stratification of patients on top of conventional risk factors. Understanding the strengths and appropriate indications of CMR, as well as the weaknesses in terms of image quality and the possible ways to overcome them, is essential for providing high-quality imaging, comprehensive information, and definitive answers in the diverse groups of patients with arrhythmias.

## Abbreviations

ACM, Arrhythmogenic Cardiomyopathy; AF, Atrial fibrillation; CMR, Cardiac Magnetic Resonance; DCM, Dilated Cardiomyopathy; HCM, Hypertrophic Cardiomyopathy; LGE, Late Gadolinium Enhancement; PVCs, Premature Ventricular Contractions; SCD, Sudden Cardiac Death; VAs, Ventricular Arrhythmias.

## Supplementary Material

Supplementary material associated with this article can be found, in the online version, at https://doi.org/10.31083/j.rcm2409252.
